# **Adverse drug reactions in persons initiated on treatment for drug-resistant tuberculosis in Kerala, India: A non-concurrent cohort study**

**DOI:** 10.1016/j.ijregi.2025.100615

**Published:** 2025-03-01

**Authors:** Raman Swathy Vaman, George Dilu Thomas, Madhanraj Kalyanasundaram, Surabhi Soman, Mathew J. Valamparampil, Rakesh Puroshothama Bhat Susheela, Manoj V. Murhekar

**Affiliations:** 1 District Hospital, Kanhangad, India; 2 FETP-MPH programme, ICMR-NIE, Chennai, India; 3 MOSC Medical College, Kolenchery, India; 4 ICMR-NIE, Chennai, India; 5 Kerala State Health Services, Thiruvananthapuram, India; 6 The Union Southeast Asia Office, New Delhi, India

**Keywords:** Adverse drug reactions, Multi-drug–resistant tuberculosis, Extensively drug-resistant tuberculosis, Treatment outcomes, India

## Abstract

**Objectives:**

We conducted a study to estimate the incidence of adverse drug reactions (ADRs) in the drug-resistant tuberculosis (DR-TB) cohort in Kerala in 2020 and describe the characteristics of the reported ADRs.

**Methods:**

A non-concurrent cohort study was conducted among all patients with DR-TB across 14 districts in Kerala from January 1, 2020 to December 31, 2020. We collected data on ADRs from patients, “Nikshay” web-portal, treatment cards, case sheets, and registers. We described ADRs by organ system, causality, severity, preventability, predictability, and seriousness of the reaction using standard tools.

**Results:**

Of the 364 persons initiated on treatment, 304 (83.5%) had at least one of the 28 listed adverse reactions, with an incidence of 27.6 ADR per 100 person-months of treatment. Gastrointestinal disorders had the highest incidence 365 per 1046 (35%). A total of 1001 of 1046 (95.7%) ADRs were non-predictable, and 405 of 1046 (39%) were definitely preventable. A total of 83 of 304 (27.3%) patients had severe ADR, and 56 of 304 (18.4%) had serious ADR. A total of 87 of 304 (28.6%) patients with ADR required interruption of the probable offending drug, with 64 of 87 (73.6%) temporary and 23 of 87 (26.4%) permanent interruptions.

**Conclusions:**

Four-fifths of all patients on therapy had at least one of the 28 listed adverse reaction and one-fifth had serious ADR. ADRs can lead to treatment interruptions. Early detection and prompt management is essential for improving treatment outcomes in patients with DR-TB.

## Highlights

• Four-fifth of all patients on drug-resistant tuberculosis therapy had an adverse reaction.
• Gastrointestinal disorders were the most common.
• A total of 27% of patients had severe adverse reactions and 18.4% had serious adverse reactions.
• A total of 39% of adverse events were definitely preventable.
• A total of 29% of patients had treatment interruptions because of adverse events.
• Only 7.4% of adverse events were reported to the Pharmacovigilance Programme.

## Introduction

Tuberculosis (TB) remains a significant global health challenge, particularly, in low- and middle-income countries. India contributes to 27% of the TB incidence and 26% of the TB-related deaths worldwide in 2022 [1]. Drug-resistant TB (DR-TB) develops when a person is infected with a *Mycobacterium tuberculosis* strain resistant to one or more of the first-line drugs used to treat TB. DR-TB can be rifampicin or isoniazid mono-resistant, multi-drug–resistant (MDR), pre-extensively drug-resistant, and extensively drug-resistant [2]. Worldwide, 0.41 million new cases of DR-TB occurred in 2022, with India contributing to more than one-fourth of the burden [1]. India has an estimated incidence of 8 DR-TB cases per 100,000 population, whereas the state of Kerala reports a much lower incidence of 1.5 per 100,000 population in 2022 [3].

Adverse drug reaction (ADR) is defined as “an appreciably harmful or unpleasant reaction, resulting from an intervention related to the use of a medicinal product, which predicts hazard from future administration and warrants prevention or specific treatment, or alteration of the dosage regimen, or withdrawal of the product [4].” The incidence of reported ADRs for DR-TB drugs ranges from 35% to 75% in India [[5], [6], [7], [8]]. Another recent study conducted in India identified that all-oral regimens had higher ADRs with a male predilection [9]. Previous program evaluations conducted in a district of Kerala identified the occurrence of ADRs in 42% of persons on treatment and was responsible for temporary or permanent treatment interruptions and non-adherence in TB management [10,11]. The health-related quality of life of persons with DR-TB was considerably lower than that of persons with drug-sensitive TB, especially in the first 6 months of therapy, with ADRs as a key factor influencing the quality of life [12]. Complete recording and analysis of adverse events and standardized data on organ class, seriousness, severity, and certainty of the association are identified as a significant research gap by the World Health Organization (WHO) [13]. Early identification, reporting and prompt management of ADRs are crucial to achieving the “WHO End TB Strategy: 2025 milestones” [14].

The WHO has established active TB drug safety monitoring and management as a TB programme component to provide active and systematic clinical and laboratory assessment of persons on drug-resistant TB treatment to detect, manage, and report suspected or confirmed drug toxicities [15]. The government of India launched the nationwide Pharmacovigilance Programme of India (PvPI) in 2010 to report ADRs by health care providers [16]. Currently, no programmatic data are available with the state of Kerala on the burden of ADRs because of DR-TB drugs and the status of ADRs. With the introduction of newer agents for DR-TB, such as bedaquiline and delamanid, which have higher toxicity profiles, it is essential to monitor ADRs to ensure patient safety and favorable treatment outcomes [17]. The present study estimates the incidence of ADRs among the cohort of persons initiated on treatment for DR-TB in Kerala in 2020, describes the characteristics of the reported ADRs, and determines the factors associated with moderate to severe ADR.

## Methods

We conducted a retrospective cohort study among all the persons initiated on treatment for DR-TB across all 14 districts in Kerala from January 1, 2020 to December 31, 2020. Line list of these patients was retrieved from “Nikshay portal” (a web-based case-based information management system for the National Tuberculosis Elimination Program). We conducted an extensive literature review, including program guidelines, to identify a list of 28 possible ADRs associated with DR-TB treatment drugs. Full details on these potential ADRs with their operational definitions can be found in Supplementary Appendix 1. We used a structured questionnaire to collect data on these 28 ADRs from persons notified as having DR-TB. For the persons who died during the study period, the data were collected from their immediate primary caregivers. ADR data were also abstracted from TB treatment cards, hospital admission records, proceedings of district-level DR-TB committee meetings, and DR-TB registers using structured data abstraction forms. In the context of a specific ADR, affirmative documentation obtained from either participant interviews or record reviews indicated a positive ADR.

To determine the risk factors for developing moderate to severe ADR (ADR that requires suspected drug be held, discontinued, or otherwise changed; or increase in length of stay or hospitalization for ADR; or requiring intensive medical care; or cause permanent harm to the patient; or directly or indirectly led to death of the patient). We compared the persons who developed moderate to severe ADR per the modified Hartwig and Seigel scale and the persons who did not develop any ADR or developed only mild ADRs during treatment. We grouped together moderate to severe ADR as the outcome variable because both are associated with treatment interruptions and unfavorable treatment outcomes [15]. We classified the ADR outcomes as recovered completely, recovered with disability, loss to follow-up (LTFU) because of ADR, and death because of ADR.

The data were collected using Epicollect 5. The quantitative variables were described by mean (SD) or median (interquartile range [IQR]) and categorical variables by proportions. The incidence of ADRs was estimated as the proportion of each ADR and ADR per person-months of treatment. The ADRs were described by organ system using the Medical Dictionary for Regulatory Activities terminology—the international medical terminology developed under the auspices of the International Council for Harmonization of Technical Requirements for Pharmaceuticals for Human Use. Causality was assessed using Naranjo's algorithm [18]. The severity and preventability of the ADRs were assessed using the modified Hartwig and Siegel scale and Schumock Thornton scale, respectively [19,20]. The seriousness of the reaction was categorized according to the Food and Drug Administration criteria [21], and predictability was determined using the Rawlins Thompsons classification by classifying the ADRs as type A (augmented) and type B (bizarre) [4]. Further details on the various criteria used for classifying the ADRs can be found in Supplementary Appendix 2.

We used generalized linear models to calculate the risk ratios with 95% confidence interval (CI) to determine the factors associated with moderate to severe ADR. Variables with *P* <0.20, plausible biological association, or evidence of significant association, as documented in earlier studies, were selected for multivariable analysis. For each variable, relevant covariates were identified as confounders by comparing the −2 log-likelihood ratio values of the models with and without the potential confounders, as per the conceptual framework based on directed acyclic graphs [22]. All statistical associations were considered significant at a two-tailed *P* <0.05. The data were analyzed using STATA ver 17 (StataCorp. 2021; Stata Statistical Software: Release 17. College Station, TX: StataCorp LLC).

### Quality assurance

We used a structured content and construct validated questionnaire to collect data on the 28 predefined ADRs with clear operational definitions to minimize interviewer bias and variation. We triangulated the data collected through interviews and abstracted from treatment cards, case records, and treatment committee minutes using Nikshay ID as the unique identifier variable to avoid duplications to ensure data completeness. All data were entered into the Epicollect5 platform, with built-in logic checks to prevent invalid entries. We used standardized tools to classify the ADRs. Two pharmacologists independently classified the ADRs, and discrepancies were arbitrated by a third senior pharmacologist. We used the κ statistics to test the inter-rater reliability among the pharmacologists. The proportion of missing data for laboratory parameters ranged from 4% to 10.7%. The missingness was confirmed to be completely at random using Little's missing completely at random test, and mean value imputation was applied to prevent biased estimates.

## Results

During 2020, Kerala notified 364 DR-TB cases across the state, with Ernakulam (n = 46, 12.6%) and Kasaragod districts (n = 41, 11.3%) reporting the maximum cases. Of them, 20 (5.5%) were health care workers. All 364 notified persons were initiated on DR-TB therapy. Table 1 shows the general characteristics of the study participants. The median (IQR) age of the participants was 48 (36-58) years. The median (IQR) interval between diagnosis and treatment initiation was 8 (4-17) days, and the median (IQR) duration of treatment was 9 (6-11) months.

**Table 1:** General characteristics of the persons notified with DR-TB in Kerala, 2020 (N = 364).

Characteristics	n	%
*Age category (in completed years)*		
≤18	9	2.5
19-45	149	40.9
46-60	136	37.4
60 above	70	19.2
*Gender*		
Male	281	77
Female	83	23
*Place of residence*		
Rural	284	78
Urban	80	22
*Scheduled tribe*	13	3.6
*Migrant worker*	9	2.5
*Occupation*		
No Job	83	22.8
Unorganized sector^a^	259	57.4
Professionals	21	5.7
Government job	13	3.6
Student	18	5
Health care worker	20	5.5
*Income*		
Below Poverty Line (Annual income<₹ 25,000 INR)	274	75.3
Above Poverty Line (Annual income≥₹ 25,000 INR)	90	24.7
*Smoking*		
Never smoker	174	47.8
Ex-smoker	41	11.3
Occasional smoker	27	7.4
Daily smoker	122	33.5
*Alcoholism*		
Never drinker	186	51.1
Ex drinker	32	8.8
Occasional drinker	80	22
Daily drinker	66	18.1
*Comorbidities*		
Hypertension	63	17.3
Diabetes mellitus	121	33.2
Coronary artery disease	20	5.5
Malignancy	10	2.8
Bronchial asthma/chronic obstructive pulmonary disease	31	8.5
Chronic kidney disease	3	0.8
Chronic liver disease	4	1.1
HIV	6	1.7
Other chronic illness on treatment	5	1.4
Previous history of any drug allergy	3	0.8
*TB Type*		
Pulmonary TB	336	92.3
Extrapulmonary TB	28	7.8
*Drug resistance type*		
INH Mono	133	36.5
MDR	218	59.9
Extensively DR	13	3.6
*Treatment regimen*		
H Mono Poly	133	36.5
Conventional MDR	30	8.2
Shorter MDR	153	42
All oral longer	42	11.5
Others^b^	6	1.8
*Patient category*		
New	221	60.7
Previously treated	143	39.3
*Place of initiating DR TB treatment*		
District TB Centre	306	84
Medical College Hospital	43	11.8
Primary Health Centre	14	3.9
Private hospital	1	0.3
*Pretreatment evaluation done*	364	100
*Admitted for minimum 1 day for pretreatment evaluation*	86	23.6

DR-TB, drug-resistant tuberculosis; MDR, multi-drug–resistant.

a Trading, farming, driving, daily wage work.

b Other individually curated regimens.

Of the 364 persons initiated on treatment, 304 (83.5%) had at least one of the 28 listed adverse reaction. Of the individuals receiving different treatment regimens, at least one of the listed ADRs were observed in 95 (71%) of those on the H mono-poly regimen, 33 (92%) on the conventional MDR regimen, 137 (89%) on the shorter MDR regimen, and 39 (93%) on the all-oral longer regimen during the study period. We identified 1046 listed ADRs during 3790 person-months of treatment, yielding an incidence rate of 27.6 ADR per 100 person-months of treatment. Considering multiple episodes of the same ADR during the follow-up, we identified 1131 episodes, giving an incidence rate of 30 ADR episodes per 100 person-months of treatment. Nausea/vomiting had the highest incidence (n = 186, 51%), followed by gastritis/abdominal pain (n = 158, 43%), and seizures had the lowest incidence (n = 1, 0.3%). Women had a slightly higher incidence of ADR (87%) than men (82%). Women had a higher incidence of all, except arthralgia, which was higher in men. The incidence of ADR was higher in individuals aged 18 years and below (89%) and above 60 years age group (83%). Table 2 shows the proportion of ADRs studied in various age and gender categories. Of these 28 predefined ADRs, none of the patients had tendinitis or tendon rupture, gynecomastia, and lactic acidosis. Other than these 28 listed ADRs, we have identified other symptoms, such as generalized weakness in 270 (74%) and myalgia in 26 (7%).

**Table 2:** Proportion of ADRs by type in persons notified with DR TB in Kerala, 2020 (N = 364).

ADR	Overall	Gender		Age group (years)			
	n (%)	Male (n = 281)	Female (n = 83)	≤18 (n = 9)	19-45 (n = 149)	46-60 (n = 136)	>60 (n = 70)
Nausea/vomiting	186 (51.1)	133 (47.3)	53 (63.4)	7 (77.8)	76 (51)	69 (50.7)	34 (48.6)
Gastritis/abdominal pain	158 (43.4)	121 (43)	37 (44.6)	4 (44.4)	59(39.6)	62 (45.6)	33 (47.1)
Generalized weakness	136 (37.4)	105 (37.4)	31 (37.4)	4 (44.4)	55 (37)	49 (36)	28 (40)
Arthralgia	86 (23.6)	68 (24.2)	18 (21.7)	2 (22.2)	33 (22.2)	38 (28)	13 (18.6)
Sleep disturbances	56 (15.4)	36 (12.8)	20 (24.1)	0	24 (16.1)	27 (20)	5 (7.1)
Headache	51 (14)	37 (13.2)	14 (17)	1 (11.1)	29 (19.5)	15 (11)	6 (8.6)
Skin pigmentation	46 (12.6)	33 (11.7)	13 (15.7)	0	20 (13.4)	19 (14)	7 (10)
Peripheral neuropathy	40 (11)	29 (10.3)	11 (13.3)	1 (11.1)	17 (11.4)	20 (14.7)	2 (3)
Taste disturbances	40 (11)	28 (10)	12 (14.5)	0	15 (10.1)	18 (13.2)	7 (10)
Vertigo	34 (9.3)	26 (9.3)	8 (9.6)	1 (11.1)	10 (6.7)	13 (9.6)	10 (14.3)
Ototoxicity	29 (8)	23 (8.2)	6 (7.2)	1 (11.1)	6 (4)	13 (9.6)	9 (13)
Tinnitus	29 (8)	24 (8.5)	5 (6)	1 (11.1)	9 (6)	13 (9.6)	6 (8.6)
Psychiatric symptoms	23 (6.3)	13 (4.6)	10 (12.1)	0	8 (5.4)	12 (8.8)	3 (4.3)
Skin rashes	22 (6)	14 (5)	8 (9.6)	1 (11.1)	10 (6.7)	7 (5.2)	4 (5.7)
Diarrhea	18 (5)	10 (3.6)	8 (9.6)	0	6 (4)	9 (6.6)	3 (4.3)
Hepatitis	17 (4.7)	12 (4.3)	5 (6)	2 (22.2)	5 (3.4)	6 (4.4)	4 (5.7)
Anemia	16 (4.4)	9 (3.2)	7 (8.4)	0	9 (6)	3 (2.2)	4 (5.7)
Thyroid abnormalities	14 (3.9)	7 (2.5)	7 (8.4)	0	5 (3.4)	5 (3.7)	4 (5.7)
Blurring of vision	14 (3.9)	9 (3.2)	5 (6)	0	5 (3.4)	9 (6.6)	0
Cardiotoxicity	10 (2.8)	5 (1.8)	5 (6)	0	3 (2)	5 (3.7)	2 (3)
Alopecia	10 (2.8)	8 (2.9)	2 (2.4)	0	2 (1.3)	5 (3.7)	3 (4.3)
Nephrotoxicity	5 (1.4)	4 (1.4)	1 (1.2)	0	1 (0.7)	3 (2.2)	1 (1.4)
Hypersensitivity reactions	5 (1.4)	4 (1.4)	1 (1.2)	1 (11.1)	3 (2)	0	1 (1.4)
Bone marrow suppression	2 (0.6)	0	2 (2.4)	0	2 (1.3)	0	0
Seizures	1 (0.3)	1 (0.4)	0	0	1 (0.7)	0	0

ADR, adverse drug reaction.

### Characteristics of the reported ADRs

#### Severity of the ADRs

**Figure 1:**
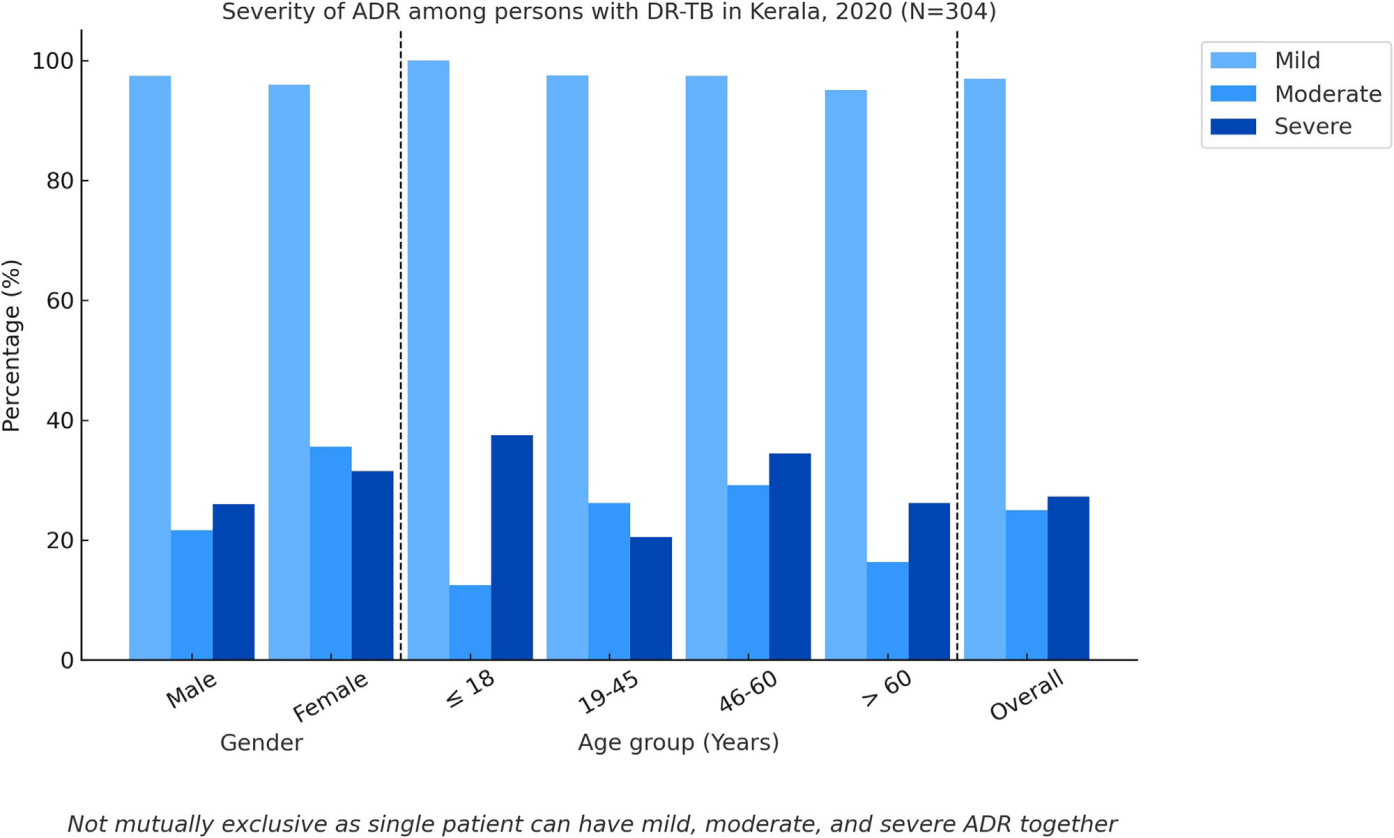
Severity of adverse drug reaction in persons with drug-resistant tuberculosis in Kerala (N = 304).

Figure 1 shows the classification of persons initiated on treatment for DR-TB by severity. Of the 304 persons having at least one of the 28 listed ADR, 295 (97%) had mild, 76 (25%) had moderate, and 83 (27.3%) had severe ADRs using the modified Hartwig and Seigel scale. Men had a higher proportion of mild ADR (n = 225, 97.4%), and women had a higher proportion of moderate (n = 26, 35.6%) and severe (n = 23, 31.5%) ADR. Only moderate ADR was found to be having a statistically significant higher proportion in women (*P* = 0.016). All persons in the age group up to 18 years (n = 8) had mild ADR, and the highest incidence of moderate and severe ADR was reported in the age groups 46-60 years (n = 33, 29.2%) and up to 18 years (n = 3, 37.5%), respectively. Of the 1046 ADRs, 803 (76.8%) were mild, 96 (9.2%) were moderate, and 147 (14%) were severe.

#### Organ system-wise classification of ADRs

**Figure 2:**
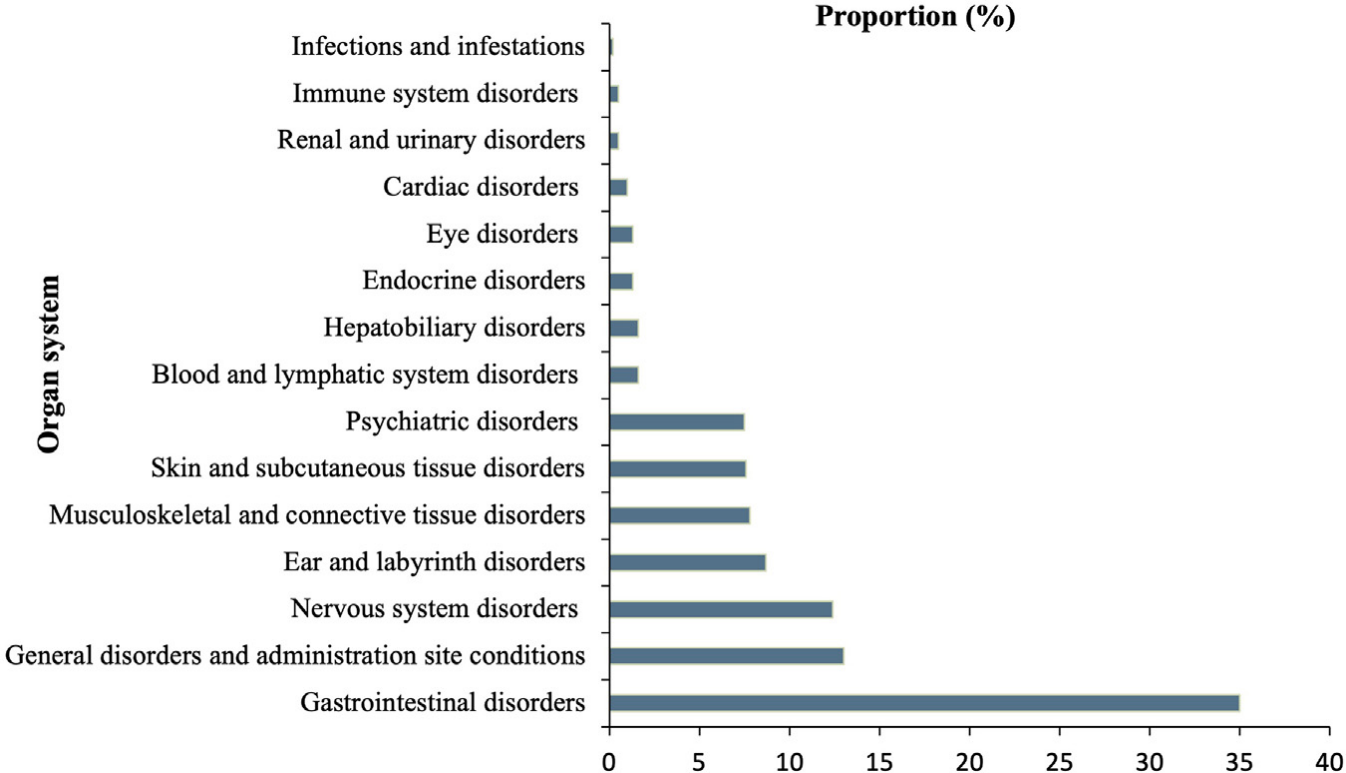
Organ system–wise classification of adverse drug reactions in persons with drug-resistant tuberculosis in Kerala (N = 1046).

Of the identified ADRs, gastrointestinal disorders had the highest incidence (n = 365, 35%), followed by general disorders and administration site conditions amounting to 135 (13%),as per Medical Dictionary for Regulatory Activities. Infections and infestations had the lowest incidence (n = 2, 0.2%). Figure 2 shows the proportion of ADRs organ system-wise.

#### Preventability of the ADRs

Of the 1046 ADRs, 405 (38.7%) were definitely preventable, 169 (16.2%) were probably preventable, and 472 (45.1%) were not preventable as per the modified Schumock Thornton criteria. Of the 405 ADRs that were definitely preventable, 365 (90%) were gastrointestinal disorders (gastritis, diarrhea, abdominal pain, or nausea-vomiting) and 40 (10%) were nervous system disorders (peripheral neuropathy). Of them, 347 (86%) were mild and 58 (14%) were moderate ADRs, as per the severity assessment. All these definitely preventable ADRs were probable ADRs, as per the Naranjo algorithm (Table 3).

**Table 3:** Definitely preventable ADRs: their organ system wise classification, severity, and causality (N = 405).

ADR	ADR events n (%)	Organ system classification^a^	Severity^b^	Causality^c^
Nausea/vomiting	189 (47)	Gastrointestinal disorders	Mild	Probable
Gastritis/abdominal pain	158 (39)	Gastrointestinal disorders	Mild	Probable
Diarrhea	18 (4)	Gastrointestinal disorders	Moderate	Probable
Peripheral neuropathy	40 (10)	Nervous system disorders	Moderate	Probable

ADR, adverse drug reaction.

a MeDRA terminology;

b Modified Hartwig-Seigel scale;

c Naranjo algorithm.

#### Predictability of the ADRs

The Rawlins-Thompson criteria classified 45 (4.3%) ADRs as type A (augmented), which is predictable, and 1001 (95.7%) as type B (bizarre), which is non-predictable or idiosyncratic.

#### Seriousness of the ADRs

Of the 304 persons with at least one of the 28 listed ADRs, 56 (18.4%) persons had serious ADRs which required prolonged hospitalization 43 (77%), permanent disability 6 (10.6%), LTFU 4 (7%), or death 3 (5.4%) and 248 (81.6%) had non-serious ADRs, as per the Food and Drug Administration criteria.

#### Causality of the ADRs

The causality assessment using Naranjo's algorithm identified 281 (27%) ADRs as possible, 765 (73%) as probable, and none were definite.

### Predictors for moderate to severe ADR

On univariate analysis, female gender, longer-duration treatment regimen, hypertension, current smoking status, low thyroid stimulating hormone (TSH) level and high blood urea level at diagnosis were significantly associated with moderate to severe ADR. After adjusting for confounders in the multivariable model, female gender (adjusted risk ratio [aRR] 1.2, 95% CI 1.1-1.3), longer-duration treatment regimen (9-24 months), oral regimen (aRR 1.7, 95% CI 1.4-2), hypertension (aRR 1.2, 95% CI 1.1-1.4), TSH at diagnosis less than 1.7 MIU/ml (aRR 1.2, 95% CI 1.1-1.3), and blood urea at diagnosis more than 18 mg/dl (aRR 1.1, 95% CI 1.1-1.3) were found to be significant predictors for developing moderate to severe ADRs during the treatment period (Table 4).

**Table 4:** Factors associated with moderate to severe ADR in persons notified with DR-TB in Kerala, 2020 (N = 364).

Exposures	Moderate to severe ADR N = 129 n (%)	No/Mild ADR N = 235 n (%)	RR (95% CI)	*P*-value	Adjusted RR (95% CI)
Age (completed years)					
>45	77 (60)	129 (55)	1 (0.9-1.2)	0.378	
≤45	52 (40)	106 (45)			
Gender					
Female	40 (31)	43 (18)	1.2 (1.1-1.3)	0.005	
Male	89 (69)	192 (82)			
Income category					
Above Poverty Line (Annual income≥25,000 INR)	37 (29)	53 (23)	1.1 (0.9-1.2)	0.195	1.1 (0.9-1.2)^a^
Below Poverty Line (Annual income<25,000 INR)	92 (71)	182 (77)			
Type of TB					
Extrapulmonary TB	14 (11)	14 (6)	1.2 (0.9-1.4)	0.093	
Pulmonary TB	115 (89)	221 (94)			
Patient category					
Previously treated	53 (41)	87 (37)	1 (0.9-1.2)	0.447	
New	76 (59)	148 (63)			
Treatment regimen					
Conventional MDR (24 months)	23 (18)	13 (5.5)	1.7 (1.4-2)	<0.001	
Shorter MDR (9 months)	61 (47)	92 (39)	1.3 (1.2-1.5)	<0.001	
All oral longer (18-20 months)	29 (23)	13 (5.5)	1.8 (1.5-2.1)	<0.001	
H monopoly (6-9 months)	16 (12)	117 (50)	1		
Diabetes mellitus					
Yes	48 (37)	73 (31)	1.1 (0.9-1.22)	0.234	
No	81 (63)	162 (69)			
Hypertension					
Yes	33 (26)	30 (13)	1.2 (1.1-1.4)	0.002	
No	96 (74)	205 (87)			
Any other comorbidity^b^					
Yes	29 (23)	40 (17)	1.1 (0.8-1.2)	0.204	
No	100 (77)	195 (83)			
Smoking					
Current smoking	40 (31)	109 (46)	0.8 (0.7-0.9)	0.004	
No current smoking	89 (69)	126 (54)			
Alcoholism					
Current alcoholic	43 (33)	103 (44)	0.9 (0.8-1)	0.050	1 (0.9-1.1)^c^
Not current alcoholic	86 (67)	132 (56)			
Body mass index at diagnosis (kg/m^2^)					
<18.5	65 (50)	128 (54)	0.9 (0.8-1.1)	0.456	
≥18.5	64 (50)	107 (46)			
Hemoglobin at diagnosis (gm/dl)					
<11	40 (31)	54 (23)	1.1 (0.9-1.2)	0.154	
≥11	89 (69)	181 (77)			
Thyroid stimulating hormone at diagnosis (mIU/ml)					
<1.7	53 (41)	58 (25)	1.3 (1.1-1.4)	0.001	1.2 (1.1-1.3)^a^
≥1.7	76 (59)	177 (75)			
Urea at diagnosis (mg/dl)					
>18	79 (61)	112 (48)	1.1 (1.1-1.3)	0.013	1.1 (1.1-1.3)^a^
≤18	50 (39)	123 (52)			

ADR, adverse drug reaction; DR-TB, drug-resistant tuberculosis; CI, confidence interval; MDR, multi-drug–resistant; RR, risk ratio.

a Adjusted for gender.

b Excluding hypertension and diabetes mellitus.

c Adjusted for gender and current smoking.

### Management of ADR, treatment interruptions, outcomes, and ADR reporting

**Figure 3:**
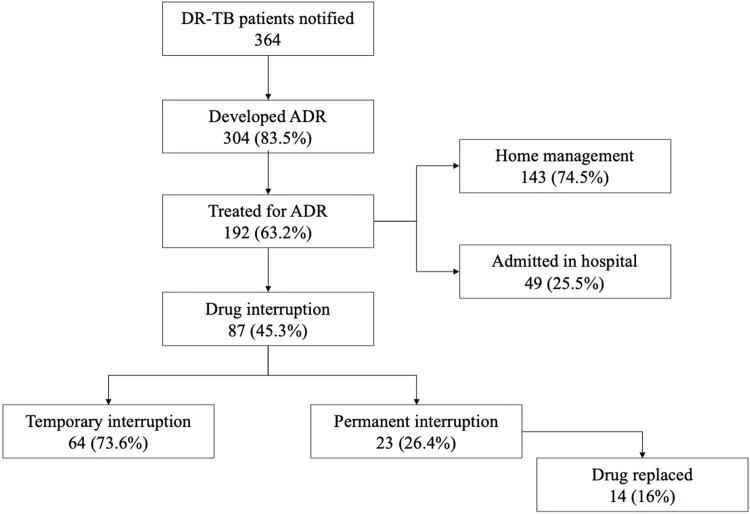
ADR management and outcomes in persons with DR-TB in Kerala (N = 364). ADR, adverse drug reaction; DR-TB, drug-resistant tuberculosis.

Of the 304 persons who had at least one of the 28 listed ADR during the treatment, 192 (63.2%) had taken any treatment for ADR symptoms, and, of them, 49 (25.5%) were admitted for management. Of the 49 persons requiring admission, 44 (89.8%) were admitted to government hospitals and 5 (10.2%) in private hospitals. The median duration of admission for ADR management was 14 days, with an IQR of 7-23 days. Of the persons who had ADR, 87 (28.6%) had an interruption of the probable offending drug by the DR-TB treatment committee, with 64 (73.6%) temporary and 23 (26.4%) permanent interruptions, with a median (IQR) interruption duration of 14 (7-14) days. Of the 23 persons with permanent interruptions, the drug was replaced in 14 (61%) persons, and 9 (39%) persons continued treatment with other drugs avoiding the offending drug. Kanamycin 7 (50%) and linezolid 5 (36%) were the most common drugs replaced. Figure 3 shows ADR management and outcomes in persons initiated on treatment for DR-TB. Considering the outcomes of ADRs, 248 (81.5%) persons were not admitted and recovered within 7 days, 15 (5%) required hospitalization and recovered fully within 7 days, 28 (9.2%) were admitted for more than 7 days and recovered fully, six (2%) were admitted for more than 7 days and recovered with disability, four (1.3%) persons were LTFU because of ADR, and three (1%) were reported deaths because of ADR. All three persons who were reported died because of ADR; the cause of death was hypersensitivity to drugs. Two died because of para amino salicylic acid and one died because of Isoniazid (INH) hypersensitivity reaction, as per clinical records and Pharmacovigilance Programme ADR reporting forms.

The treatment outcomes of persons notified as DR-TB initiated on treatment were 248 (68.2%) cured, 50 (13.7%) treatment completed, 4 (1.1%) not evaluated, 4 (1.1%) treatment failure, 19 (5.2%) LTFU, and 39 (10.7%) died during the treatment.

Of the 1046 ADRs, only 77 (7.4%) were reported to the PvPI and had ADR reporting forms at the treating facility. Of the reported ADRs, 54 (71%) were of high severity ADRs.

## Discussion

We found that eight of 10 patients had at least one of the 28 listed ADR during the course of treatment, with an incidence rate of 27.6 per 100 person-months of treatment. Gastrointestinal disorders were the most commonly reported. A total of 83 (27.3%) persons with ADR had severe ADR and 56 (18.4%) had serious ADR and less than 5% were preventable.

Our study identified a high incidence of ADR in persons notified as having DR-TB. The authors intended to capture the ADRs of entire cohort of patients started on treatment for the particular year. Because the DR-TB treatment duration ranges from 6 months to 2 years, we have selected 2020 as our reference year because we expected the patients who were initiated treatment by December 3, 2020 will have attained treatment outcomes by December 2022 and can be included in our analysis. A hospital-based study conducted in North India by Kumari *et al.* [23] identified the incidence of ADRs as high as 87% in persons notified as having DR-TB during treatment. Several other studies conducted in India have estimated the incidence of ADR in persons with DR-TB varying from 34% to 75% [6,7,[24], [25], [26], [27], [28]]. The high incidence of DR-TB identified in the present study may be because of the introduction of newer agents with a higher toxicity profile and high morbidity profile of the study population. The difference in ADR incidence reported could be attributed to differences in the treatment regimen, opinion differences of patients and physicians in ADR reporting, treatment adherence, default rate, and varying ADR management in different settings.

Nausea/vomiting and gastritis, the most common symptoms, were present in the range of 20-70% among various treatment cohorts in other parts of the world as well [6,[23], [24], [25], [26],[28], [29], [30], [31]]. Our study has identified a high incidence of sleep disturbances among the participants, which contrasted with previous evidence from India [6]. A study from China on the sleep quality of persons on treatment for TB reported sleep disturbances to varying degrees in 70% of them [32]. Our study reports a lower incidence of psychiatric symptoms (depression, anxiety disorder, psychosis) than a recent study published in India [33]. A systematic review and meta-analysis by Alene *et al.* [12] has estimated that 25%, 24%, and 10% of persons notified as having DR-TB experience depression, anxiety, and psychosis during treatment, respectively. As per the state policy, all persons on treatment are evaluated for psychological well-being and the lower incidence reported may be because of the support provided to these persons by the health and local self-governments, reducing social, financial, and psychological stressors.

The incidence of ADR was higher in those under 18 years old and above 60 years old. Most of the previously published studies report the highest incidence of ADR among the 30-60 years age group. Higher incidence in those older than 60 years may be because of the higher prevalence of non-communicable diseases, such as hypertension and diabetes mellitus, in the study population, which may precipitate the occurrence of ADRs. The high incidence of ADR in those younger than 18 years is also a matter of concern because it interferes with education, leading to dropouts, and the disabilities associated with the ADRs grossly affect the quality of life for these young persons [34]. Women reported a higher proportion of ADRs in our study. This is in accordance with previously published studies from India and Pakistan, which had a higher incidence among women on DR-TB treatment [28,30,31].

Our study identified the incidence of severe ADRs as 14%, similar to a study conducted in Telangana [28]. Other studies from India have identified severe ADR of 23% in persons notified as having DR-TB [6,26]. The elevated incidence documented could be attributed to the underreporting of mild ADRs, given that these investigations primarily concentrated on individuals seeking medical attention in hospitals and, consequently, predominantly encompassed ADRs falling within the moderate and severe categories.

Our present study reports a higher proportion of possible preventable ADRs than other studies, which reported non-preventable ADRs ranging from 30% to 85% [6,28]. We have identified 39% of these reported ADRs (gastrointestinal and nervous system disorders) as definitely preventable because there are definite treatment in the form of proton pump inhibitors, H_2_ receptor blockers, and pyridoxine for the previously mentioned ADRs. However, we encountered challenges in pinpointing the exact causality of these ADRs because of the complexity of the treatment regimens. All patients were on multiple drugs as part of their MDR-TB therapy, making it difficult to attribute specific reactions to individual medications. Because these ADRs are very common, the clinicians should think about these ADRs and start appropriate prophylactic treatment at the initiation of therapy. A high proportion of mild gastrointestinal ADRs can be given drug prophylaxis to prevent or reduce the symptoms. Two studies conducted in India, which assessed the causality using Naranjo's algorithm, have reported definite ADRs of 12.3% and 2.8%, probable ADRs of 34.7% and 67.8%, and possible ADRs of 53% and 29.3%. We report a higher proportion of probable ADR, and none was definite because the symptom combinations were not definitively conclusive of the fixed drug combinations of DR-TB drugs.

Female gender, longer oral DR-TB regimens, hypertension, TSH value below 1.7 mIU at diagnosis, and blood urea level more than 18 mg/dl at diagnosis were associated with the development of moderate to severe ADR. Our study identified a higher risk of moderate to severe ADR among women, similar to a prospective cohort study conducted in India [35]. This could be attributed to the gender-related differences in pharmacokinetic, immunologic, and hormonal factors. New-generation, shorter MDR and all-oral longer regimens have better efficacy and high toxicity profile [36,37]. We report a strong association between these regimens and ADRs. Many studies worldwide have identified comorbidities as a risk factor for ADR during TB treatment [35,38,39]. Our study identified hypertension as a risk factor for developing ADRs, consistent with a recent prospective cohort study conducted in India [35].

We report TSH and blood urea values at diagnosis as a predictor for moderate to severe ADR. Previously, laboratory parameters such as hypoalbuminemia, anemia, hyponatremia, and lymphopenia were used to predict ADRs in patients with TB on treatment [40]. The role of TSH and blood urea at diagnosis as ADR predictors needs to be explored further.

The drug interruptions because of ADR reported in our study are similar to those published in India, where the offending drug was interrupted in 25% of patients [26]. In a recent study conducted in India on persons on bedaquiline-containing regimens, ADRs were the most common reason for interruption observed in 81.4% of the temporary interruption group and 97.1% of the permanent interruption group [41].

Kanamycin and linezolid were the commonly replaced drugs in the 2020 cohort in Kerala. Another study published in Kerala reported kanamycin, cycloserine, and ethambutol as the commonly replaced drugs [6].

In our study, less than two-fifths of the patients with ADR required treatment, and a quarter were admitted for managing ADRs. This implies that most ADRs were mild, which can be managed by domiciliary care if diagnosed early. Hence, the sensitization of primary care providers at the peripheral level in the early detection and management of ADR is of prime importance.

As identified by our study, LTFU because of ADR is consistent with other studies from India and similar settings, attributing ADR as an important factor for LTFU [[42], [43], [44]].

From our study, less than one-tenth of the ADRs have been reported to the PvPI. This is of serious concern because these reported data will be used to characterize the types of adverse reactions, assess the safety of the treatment, and inform future policy on the use of these medicines. This data gap from low- and moderate-income countries with the maximum TB burden hinders patient safety monitoring and needs to be addressed immediately.

In this context, we would like to highlight that patient-centered pharmacovigilance is crucial for enhancing drug safety and improving health outcomes. It shifts the focus from merely monitoring drug effects to understanding the patient experience with ADRs. By actively involving patients in pharmacovigilance, we can improve drug safety, enhance patient care, and promote a culture of safety and transparency in the health care industry [45].

## Strengths and limitations of the study

### Strengths

To the best of our knowledge, this is one of the first studies in India to estimate the incidence of ADR among the entire cohort of persons notified as DR-TB in a state after introducing the newer regimens. We have captured an exhaustive list of 28 ADRs among the patients on treatment, treatment interruptions and outcomes of these ADRs, which was not previously done. Given that these drugs are standardized and prescribed as part of national treatment guidelines, our findings have broader relevance beyond Kerala. Although regional variations in genetic, nutritional, and health care factors may influence ADR profiles, the overall patterns observed in our study can provide important insights applicable to other parts of the country.

### Limitations

Information bias could arise because of the reliance on patient-reported symptoms and clinician-documented ADRs. Self-reported ADRs are subjective and may vary based on an individual's perception of symptoms, health literacy, and willingness to disclose certain conditions. Clinicians’ documentation practices may also differ, leading to underreporting or misclassification of ADRs, particularly, mild or transient symptoms. To mitigate this, the study used data triangulation from patient interviews, treatment cards, and hospital records, which likely reduced but did not eliminate this bias. Future studies could benefit from real-time ADR recording using digital tools to minimize inconsistencies in reporting.

Given the retrospective nature of the study, participants may not accurately recall the onset, severity, or duration of ADRs, especially those occurring early in the treatment course. This could lead to underestimation of the true incidence of ADRs. The issue was partially addressed by corroborating patient-reported data with medical records. However, recall bias remains a limitation, particularly, for patients who experienced mild symptoms not requiring medical attention.

During the data collection period, approximately 10% of the patients had died, and we collected data from their immediate primary caregivers, which may induce an information bias. For deceased patients, caregiver interviews may have been subject to recall bias, with potential inaccuracies in recalling the timing, sequence, or specific symptoms preceding death. We conducted a subgroup analysis for ADR reporting among the living and expired patients, and no significant differences existed in ADR reporting between these two groups. There can be an ascertainment bias because patients who had visited the health facility or were admitted for ADR may have been evaluated thoroughly to find out more ADRs, and, hence, more reporting would have been done for these patients. Because this retrospective study was done on persons initiated on treatment in 2020 and complete coverage of newer drugs were attained in the state by 2022, few adverse reactions because of the change in drug regimen may not have been captured in the present study. Given the retrospective nature of our study, coupled with the complex medication regimens of our patient population with individual drugs having overlapping ADR profiles, establishing direct causal relationships between specific drugs and ADRs were not done. The missing data in the laboratory parameters could have biased the estimates for risk factors. However, they were missing completely at random and we imputed the mean values to get the unbiased estimates.

## Conclusion

DR-TB requires prolonged and complex treatment and was associated with a spectrum of adverse reactions. Four-fifth of all patients on therapy had at least one of the 28 listed adverse reaction, and one-fifth had serious ADR. Two-fifth of these ADRs were definitely preventable. Only less than one-tenth of the ADRs were reported, and three-fourths were successfully treated at home. ADRs were associated with treatment interruptions, LTFU, and unfavorable treatment outcomes. Early detection of ADRs at the primary care level and their prompt management is essential for improving treatment outcomes in patients with DR-TB. Continuous pharmacovigilance and active ADR surveillance at the national level is crucial for early detection and management of ADRs, ensuring patient safety, and optimizing treatment adherence.

## CRediT authorship contribution statement

**Raman Swathy Vaman:** Conceptualization, Methodology, Software, Validation, Formal analysis, Investigation, Data curation, Writing – original draft, Writing – review & editing, Visualization. **George Dilu Thomas:** Methodology, Validation, Formal analysis, Data curation, Writing – original draft, Writing – review & editing. **Madhanraj Kalyanasundaram:** Conceptualization, Methodology, Formal analysis, Data curation, Writing – original draft, Writing – review & editing. **Surabhi Soman:** Methodology, Validation, Formal analysis, Data curation, Writing – original draft, Writing – review & editing. **Mathew J. Valamparampil:** Methodology, Validation, Formal analysis, Data curation, Writing – original draft, Writing – review & editing. **Rakesh Puroshothama Bhat Susheela:** Conceptualization, Methodology, Formal analysis, Data curation, Writing – original draft, Writing – review & editing. **Manoj V. Murhekar:** Conceptualization, Methodology, Formal analysis, Data curation, Writing – original draft, Writing – review & editing, Supervision.

## Declarations of competing interest

The authors have no competing interests to declare.

## Appendix Supplementary materials

Image, application 1
