# Supplementary material for: Adverse drug reactions in persons initiated on treatment for drug-resistant tuberculosis in Kerala, India: A non-concurrent cohort study
Source: IJID Reg. 2025 Mar 1;15:100615. doi: 10.1016/j.ijregi.2025.100615 (PMC11979944; doi:10.1016/j.ijregi.2025.100615)
Supplement: Supplementary file 1 [file mmc1.docx]

**Supplementary appendix 1**

**Possible adverse events due to DR-TB drugs**

| **Si No** | **Adverse events** | **Common possible drugs** |
| --- | --- | --- |
| 1 | QT prolongation (QTcF > 450 ms in Males / 470 in Females) | Bdq, FQ, Cfz |
| 2 | Rash, allergic reaction and anaphylaxis | Any drug |
| 3 | Nausea & vomiting | Eto, PAS, Z, E, Bdq |
| 4 | Gastritis & Abdominal pain | PAS, Eto, Cfz, Lzd, FQs, H, E, and Z |
| 5 | Diarrhoea and or flatulence | PAS, Eto |
| 6 | Hepatitis AST/ALT elevation >= 5x UNL with normal bilirubin OR AST or ALT elevation >= 3x UNL accompanied by bilirubin >= 2x UNL  OR Symptomatic patient | Z, H, R, Eto, PAS, Bdq |
| 7 | Giddiness | Am, Eto, FQ and/or Z |
| 8 | Hypothyroidism (TSH <0.5 mIU/L) | Eto, PAS |
| 9 | Arthralgia | Z, FQ, Bdq |
| 10 | Peripheral Neuropathy | Lzd, Cs, H, Am, FQ, rarely Eto, E |
| 11 | Headache | Bdq, Cs |
| 12 | Depression (as mentioned in case records) | Cs, FQ H, Eto |
| 13 | Psychotic symptoms (as mentioned in case records) | Cs, H, FQ |
| 14 | Suicidal ideation (as mentioned in case records) | Cs, H, Eto |
| 15 | Seizures | Cs, H, FQ |
| 16 | Tendonitis and tendon rupture | FQ |
| 17 | Vestibular toxicity (Tinnitus & Dizziness) | FQ, H, Eto, Am, Cs, Lzd |
| 18 | Optic neuritis | E, Lzd, Eto, Cfz, H, S |
| 19 | Metallic taste | Eto, FQ |
| 20 | Gynecomastia | Eto |
| 21 | Alopecia | H, Eto |
| 22 | Superficial fungal infection and thrush | FQ |
| 23 | Dysglycemia and Hyperglycemia | Eto |
| 24 | Hematological abnormalities (Leukopenia, thrombocytopenia, anemia, red cell aplasia,  coagulation abnormalities and eosinophilia) | Lzd |
| 25 | Nephrotoxicity (Symptoms and/or signs of renal impairment (oliguria,  anuria, puffiness of face, pedal oedema) and or RFT derangement) | Am |
| 26 | Hearing loss (Threshold > 40 dB at 4000- 8000 Hz/ Threshold > 40 dB at 2000- 4000 Hz/ Threshold > 40 dB at 1000- 8000 Hz) | Am |
| 27 | Hypokalemia (Serum K <3.6 mmol/L) & Hypomagnesemia (Serum Mg <1.46 mg/dL) | Am |
| 28 | Lactic acidosis | Lzd |

Bdq-Bedaquiline

FQ- Flouroquinolones

Cfz- Clofazamine

Eto- Ethionamide

PAS- Para aminosalicyclic acid

Z- Pyrazinamide

E- Ethambutol

H- Isoniazid

Lzd- Linezolid

R- Rifampicin

Am- Amikacin

Cs- Cycloserine

**Supplementary appendix 2**

**ADR characteristics, tools and classification**

| **Organ system wise classification** | **Medical dictionary for regulatory activities (MeDRA)** | **Classification**   1. Blood and lymphatic system disorders 2. Cardiac disorders 3. Congenital, familial and genetic disorders 4. Ear and labyrinth disorders 5. Endocrine disorders 6. Eye disorders 7. Gastrointestinal disorders 8. General disorders and administration site conditions 9. Hepatobiliary disorders 10. Immune system disorders 11. Infections and infestations 12. Injury, poisoning and procedural complications 13. Investigations 14. Metabolism and nutrition disorders 15. Musculoskeletal and connective tissue disorders 16. Neoplasms benign, malignant and unspecified (including cysts and polyps) 17. Nervous system disorders 18. Pregnancy, puerperium and perinatal conditions 19. Psychiatric disorders 20. Renal and urinary disorders 21. Reproductive system and breast disorders 22. Respiratory, thoracic and mediastinal disorders 23. Skin and subcutaneous tissue disorders 24. Social circumstances 25. Surgical and medical procedures 26. Vascular disorders 27. Product issues |
| --- | --- | --- |

| **Causality** | **Naranjo’s Algorithm** | **Score** | **Interpretation** |
| --- | --- | --- | --- |
| \| **Question** \| **Yes** \| **No** \| **Do Not Know** \| \| --- \| --- \| --- \| --- \| \| 1.Are there previous conclusive reports on this reaction? \| +1 \| 0 \| 0 \| \| 2.Did the adverse event appear after the suspected drug was administered? \| +2 \| -1 \| 0 \| \| 3.Did the adverse event improve when the drug was discontinued or a specific antagonist was administered? \| +1 \| 0 \| 0 \| \| 4.Did the adverse event reappear when the drug was readministered? \| +2 \| -1 \| 0 \| \| 5.Are there alternative causes that could on their own have caused the reaction? \| -1 \| +2 \| 0 \| \| 6.Did the reaction reappear when a placebo was given? \| -1 \| +1 \| 0 \| \| 7. Was the drug detected in blood or other fluids in concentrations known to be toxic? \| +1 \| 0 \| 0 \| \| 8. Was the reaction more severe when the dose was increased or less severe when the dose was decreased? \| +1 \| 0 \| 0 \| \| 9. Did the patient have a similar reaction to the same or similar drugs in any previous exposure? \| +1 \| 0 \| 0 \| \| 10. Was the adverse event confirmed by any objective evidence? \| +1 \| 0 \| 0 \| | | Total Score  ≥9 | **Definite**. The reaction (1) followed a reasonable temporal sequence after a drug or in which a toxic drug level had been established in body fluids or tissues, (2) followed a recognized response to the suspected drug, and (3) was confirmed by improvement on withdrawing the drug and reappeared on reexposure |
|  |  | Total Score  5 to 8 | **Probable**. The reaction (1) followed a reasonable temporal sequence after a drug, (2) followed a recognized response to the suspected drug, (3) was confirmed by withdrawal but not by exposure to the drug, and (4) could not be reasonably explained by the known characteristics of the patient’s clinical state |
|  |  | Total Score  1 to 4 | **Possible**. The reaction (1) followed a temporal sequence after a drug, (2) possibly followed a recognized pattern to the suspected drug, and (3) could be explained by characteristics of the patient’s disease. |
|  |  | Total Score  ≤0 | **Doubtful**. The reaction was likely related to factors other than a drug. |

| **Severity** | **Modified Hartwig and Siegel scale** |  |
| --- | --- | --- |
| Level 1 | An ADR occurred but required no change in treatment with the suspected drug. | **Mild** |
| Level 2 | The ADR required that treatment with the suspected drug be held, discontinued, or otherwise changed. No antidote or other treatment requirement was required. No increase in length of stay (LOS) |  |
| Level 3 | The ADR required that treatment with the suspected drug be held, discontinued, or otherwise changed.  AND/OR  An Antidote or other treatment was required. No increase in length of stay (LOS) | **Moderate** |
| Level 4 | Level 4a - Any level 3 ADR which increases length of stay by at least 1 day.  Level 4b - The ADR was the reason for the admission |  |
| Level 5 | Any level 4 ADR which requires intensive medical care | **Severe** |
| Level 6 | The adverse reaction caused permanent harm to the patient |  |
| Level 7 | The adverse reaction either directly or indirectly led to the death of the patient |  |

| **Preventability** | | **Modified Schumock and Thornton scale** | |
| --- | --- | --- | --- |
| **Questions for assessment of preventability** | | | |
| **Definitely preventable** | | | |
|  | Was there a history of allergy or previous reactions to the drug? | | **Definitely preventable if any one or more answer is yes** |
|  | Was the drug involved inappropriate for the patient’s clinical condition? | |  |
|  | Was the dose, route or frequency of administration inappropriate for the patient’s age, weight or disease state? | |  |
|  | Was a toxic serum drug concentration (or laboratory monitoring test) documented? | |  |
|  | Was there a known treatment for the Adverse Drug Reaction? | |  |
| **Probably preventable** | | | |
| 6. | Was required Therapeutic drug monitoring or other necessary laboratory tests not performed? | | **Probably preventable if any one or more answer is yes** |
| 7. | Was a drug interaction involved in the ADR? | |  |
| 8. | Was poor compliance involved in the ADR? | |  |
| 9. | Were preventative measures not prescribed or administered to the patient? | |  |
| **Not preventable** | | | |
| If all above criteria not fulfilled | | | **Not preventable if all answers are No** |

| **Predictability** | **Rawlins -Thompsons criteria** | **Type A or Augmented reactions or Predictable** occur in response to drugs given at therapeutic dose and are the result of an abnormal response of an otherwise normal pharmacological effect of a certain medicine. They are common but unlikely to be associated with a fatal event.  **Type B or idiosyncratic reactions or Unpredictable** are unrelated to the pharmacological effect or the dosage of the drug and are often fatal. |
| --- | --- | --- |
| **Seriousness of the reaction** | **Food and Drug Administration (FDA) criteria** | **Serious ADR** An adverse event or suspected adverse reaction is considered "serious" if, in the view of either the investigator or sponsor, it results in any of the following outcomes: Death, a life-threatening adverse event, inpatient hospitalization or prolongation of existing hospitalization, a persistent or significant incapacity or substantial disruption of the ability to conduct normal life functions, or a congenital anomaly/birth defect. Important medical events that may not result in death, be life-threatening, or require hospitalization may be considered serious when, based upon appropriate medical judgment, they may jeopardize the patient or subject and may require medical or surgical intervention to prevent one of the outcomes listed in this definition |
